# Neuronal physiology of amygdala neurons in the context of injury and pain

**DOI:** 10.1016/j.ynpai.2025.100190

**Published:** 2025-06-27

**Authors:** Blesson K Paul, Maria Isabel Nunez-Ordaz, Joseph R. Samuel, Benedict J. Kolber

**Affiliations:** Department of Neuroscience and Center for Advanced Pain Studies, University of Texas at Dallas, Richardson, TX 75080, United States

**Keywords:** Amygdala, Pain, Central Amygdala, Electrophysiology, Neuronal Physiology, Synaptic Plasticity, Basolateral Amygdala, Parabrachial Nucleus, Prefrontal Cortex, Cognitive-Impairment, Pain-mediated synaptic plasticity, Pain-mediated membrane excitability, Nociception, Lateralization, Calcitonin Gene Related Peptide Receptor, Protein Kinase C δ, Somatostatin, Corticotrophin Releasing Factor, Opioid receptors

## Abstract

The amygdala integrates polymodal information including nociceptive stimuli. It is implicated as a key node in regulating both sensory-discriminative and emotional-affective aspects of pain and central sensitization. While central sensitization mechanisms in persistent pain are not completely understood, studying the neuronal properties exhibited by the amygdala neurons within their functional context, in this case, nociception, is important. Such studies can shed light on the behavior-modulating potential of the amygdala. In the last twenty years, multiple laboratories have begun the process of dissecting the cell-type specific activity involved in amygdala function. This review surveys these electrophysiological properties of neurons from different amygdala nuclei, cell types and circuitry studied so far, in the context of nociception and injury. A population-level accounting of these cell types provides greater insight into identifying specific targets to develop interventions for pain-mediated affective conditions.

## Highlights

• Current understanding of nociceptive processing within the amygdala.
• Electro-responsive properties of amygdala cell types under normal conditions and post-injury states.
• The contribution of opioid receptors to synaptic transmission and membrane excitability within the amygdala.
• Known amygdala cell types and their physiology, with a focus on those yet to be determined in the context of pain.

## 1 Introduction

Nociception and pain are complex processes that integrate peripheral sensory activity with cognition and emotion. Providing a protective function either through primitive spinal reflexes or complex conscious and subconscious supraspinal responses, nociception is critical to survival (Verpoorten et al. 2006). The features of noxious mechanical, thermal, and chemical stimuli are transduced as inward generator currents through nociceptors. These features include the intensity, spatial patterns and temporal patterns of stimuli. These signals are carried by fast conducting thinly myelinated Aδ/Aβ or slowly conducting unmyelinated C fibers to the soma of sensory ganglia such as dorsal root ganglia (DRG), trigeminal ganglia (TG), or nodose ganglia (Kupari et al. 2019). The information is then relayed to the central nervous system (CNS) through second order neurons in the spinal cord (Woolf et al. 2007). These signals are further processed in higher centers of the brainstem and brain which results in appropriate behavioral responses such as withdrawal from or avoidance of the unpleasant stimulus. These behavioral responses then serve to limit further damage, stimulate healing, and provide important signals for learning and memory associated with an injury (Walters, 1994).

Following repeated noxious stimulation, nociceptor activation thresholds often decrease and subsequent responses to nociceptive stimuli are enhanced. The enhancement of neuronal function in the CNS circuitry following injury or repeated nociceptive stimulation is referred to as ‘central sensitization’ (Woolf, 1983, Latrémolière and Woolf, 2009). The “Kyoto protocol” from the International Association for the Study of Pain (IASP) defines central sensitization as the “increased responsiveness of nociceptive neurons in the CNS to their normal or subthreshold input” (Loeser and Treede, 2008). Under normal circumstances, central sensitization is an activity-dependent adaptive mechanism (Ji et al. 2003). The sensitized state is typically reversed after the source of the injury has ceased triggering nociceptor activation and the site of injury has healed. The neuronal activation threshold returns to baseline, receptive fields contract, and thresholds for pain decrease. However, in conditions where the individual is in a persistent state of pain, the sensitized state continues beyond the healing of the initial injury.

Injury-induced inflammation or neuronal disorder-mediated neuropathic conditions sensitize the pre- and post-synapses and modulate the neuronal excitability of nociceptive circuits throughout the nervous system. This includes sensory ganglia (Drew et al. 2001), dorsal horn neurons (Aby et al. 2018), brain stem (Kim et al. 2012), thalamico-cortical structures (Guilbaud et al., 1986, Burstein et al., 2010), and the limbic system (Woolf, 1983, Mannion et al., 1999, Ji et al., 2003, Delcroix et al., 2003, Han et al., 2004, Park et al., 2016). Maladaptive plasticity in any nociceptive circuit facilitates persistent neuronal hyperexcitability and the reduction of inhibitory control. The resulting amplification in nociceptive transmission leads to various pathologies where pain perception is exaggerated to mildly noxious stimuli (hyperalgesia) or to previously innocuous stimuli (allodynia) (Neugebauer et al., 2003, Kang et al., 2020). Persistent or chronic pain after healing or in the absence of injury is generally considered maladaptive since there are no obvious protective or reparative benefits. There are other opinions that suggest that chronic pain induces hypervigilance during a post-injury vulnerability state, which could be advantageous for injury avoidance and survival (Walters et al. 2019). Nonetheless, chronic pain does significantly affect a normal and a healthy lifestyle.

Chronic pain conditions often contribute to frustration, severe depression, and anxiety and may even lead to substance abuse and suicide (Dworkin and Gitlin, 1991, McWilliams et al., 2003, Kroenke et al., 2013). The ramifications of living with chronic pain conditions include the general toll on the healthcare system and substantial economic costs (Henschke et al. 2015). In this review, we take the perspective that in the study of pain, the continual dissection of cellular mechanisms using electrophysiological methods is important for formulating effective intervention for pain disorders. We take this perspective because our fundamental understanding of the neuronal function has come from electrophysiology.

This perspective is well illustrated by classic studies of capsaicin/TRPV1 (transient receptor potential vanilloid type 1) channels. Capsaicin, the pungency causing ingredient of hot chili peppers has been known to evoke a painful thermal perception followed by a desensitization phase in peripheral sensory neurons (Jancsó et al., 1967, Nagy et al., 2004). Linking capsaicin actions to target receptor(s) has opened up relevant insight into peripheral sensory neuron fundamental biology. The primary receptor for capsaicin, the TRPV1 protein, was famously characterized using electrophysiological studies by exogenously expressing it in Xenopus oocytes, HEK293 cells and cultured DRG neurons (Caterina et al., 1997, Caterina et al., 2000, Babes et al., 2002, Raisinghani and Premkumar, 2005, Cesare et al., 1999). TRPV1 is an outwardly rectifying, non-selective cation channel with high calcium permeability. Beyond multiple over-the-counter capsaicin formulations for pain, in 2009, capsaicin (8 % patch) was approved as a TRPV1 agonist found to relieve neuropathic pain conditions such as post-herpetic neuralgia and diabetic peripheral neuropathy (Anand and Bley, 2011). Several studies utilizing electrophysiological methods have identified more than 30 endogenous (Benítez-Angeles et al., 2020) and more than 136 exogenous (Abbas, 2020) agonists that modulate TRPV1 activity (Xia et al. 2010). Overall, the study of the physiology of TRPV1 channels has led to the development of several synthetic drugs with both agonistic and antagonistic mechanisms of action that are being explored to treat migraine, post-surgical, musculoskeletal, neuropathic, arthritic, and dental pain (Garami et al., 2020, Iftinca et al., 2020).

While the study of nociceptor physiology in the peripheral nervous system (PNS) has yielded several actionable insights, this same process is in its relative infancy in the CNS. The challenges of developing CNS-targeted therapies are well known. These challenges include the blood–brain barrier permeability, side-effects, inaccessibility to each manipulation, and redundancy of single proteins across varied cognitive functions. However, it is important to consider how nociceptive stimuli are processed at the synaptic level in the CNS under both acute and chronic pain conditions. In this review, we focus our analysis on physiological studies within the amygdala, one of the most important limbic system nociceptive information processing regions.

The involvement of amygdala has been shown in functional imaging studies conducted in humans, in experimental and in clinical pain conditions (Simons et al. 2012). Chronic pain conditions are often comorbid with affective disorders such as depression, stress, and anxiety. A recent *meta*-analysis found that the right central nucleus of the amygdala (CeA) was the most common brain structure identified when pain precedes affective disorders (Zheng et al. 2022). As an integrator of polymodal sensory and nociceptive information, the amygdala consists of neurons that are highly plastic (McKernan and Shinnick-Gallagher, 1997, Ikeda et al., 2007; Gonçalves et al., 2008; Pape and Pare, 2010) suggesting that experience-mediated modulations in the amygdala neurons can contribute to the emotional-affective aspects of pain (Neugebauer, 2015, Thompson and Neugebauer, 2017).

## 2 Amygdala nociceptive circuitry

The amygdala receives nociceptive information through two primary pathways. The first is a highly processed multimodal sensory information stream originating from the posterior thalamic nuclei and cortical areas (i.e., the spinothalamic tract). Within this spinothalamic stream, nociceptive signals are received by the lateral amygdala (LA) and the baso-lateral amygdala (BLA) complex (McDonald, 1998, Lanuza et al., 2008), which subsequently project to the amygdala's central nucleus (CeA) (LA/BLA-CeA) or outside the amygdala. The second is the spinal cord-parabrachial nuclei (PBN)-amygdala pathway (spino-parabrachio-amygdala) (Bernard and Besson, 1990, Gauriau and Bernard, 2002).

The spino-parabrachio-amygdala pathway directly projects to the CeA (Hunt and Mantyh, 2001). A recent study showed that the three major subdivisions of CeA, namely the capsular region (CeC), lateral division (CeL) and the medial division (CeM), receive monosynaptic excitatory inputs from the PBN (Li and Sheets, 2020). These sub-divisions also receive excitatory inputs from the LA and BLA (Zhu and Pan, 2004) along with feedforward inhibitory inputs from the intercalated cells (ITC) (Royer et al. 1999). The ITC mass consists of inhibitory neurons that is interposed between LA-BLA and CeA. The ITC receives glutamatergic inputs from the LA-BLA and mPFC (medial prefrontal cortex) (Pinard et al. 2012). The CeC has been aptly termed the “nociceptive amygdala” as the convergence of LA-BLA and PBN inputs onto the CeA divisions (PBN-CeA) enables it to respond to both noxious and innocuous stimuli (Gauriau and Bernard, 2002, Neugebauer et al., 2004).

The anatomical terminologies used in the literature identifying different sub-nuclei of amygdala components have several variations that have evolved over time. In this review we have used consistent terms to denote the different amygdalar nuclei as LA, BLA, CeC, CeM and CeL. Some studies use the acronym BA (basal amygdala) to denote BLA. BA is synonymous with BLA. Throughout the review we mention the nuclei that are located within the amygdala capsule (ventral extension of external capsule as a fork) as LA (dorsally located) and BLA (ventrally located). In some instances, we refer to them individually as LA and BLA, however, for functional reason we mostly categorize LA and BLA together and refer them collectively as the LA-BLA complex. The sub-divisions that are located further medial to the LA-BLA complex are collectively called the CeA. Within the CeA, the capsular region is sometimes termed as latero-capsular nuclei (CeLC), especially in older papers, and is synonymous to CeC. However, some papers refer to the CeLC in the sense that comprises of both CeC and CeL. Such a combined designation may be a pragmatic approach due to the difficulty in clearly demarcating the boundary between CeC and CeL, especially in the anterior sections. Here we use CeC, CeL and CeM as the standard terms throughout this review to denote the capsular, lateral and medial region of the central amygdala, respectively.

Beside the LA/BLA and CeA, the amygdalar complex consists of the prominent structures such as the basomedial amygdala (synonymous with accessory basal nucleus), medial amygdala (MeA) and the superficial cortex-like group which corresponds to the cortical amygdala (CoA) nuclei (Sah et al., 2003, Beyeler and Dabrowska, 2020, McDonald, 1998). The basomedial nuclei is located ventral to the BLA. It is implicated in anxiety like behaviors in rats and autonomic function such as heart rate regulation (Mesquita et al, 2016). MeA, located lateral to the optical tract, receives olfactory and vomeronasal inputs and has been implicated in social cues, reproductive behavior and innate fears (Butler et al., 2012, Adekunbi et al., 2018). The superficial cortex-like group is located on the brain's surface, and its neuronal structure is similar to that of the adjacent olfactory cortex. This group consists of the following sub-nuclei. The anterior subdivision of the cortical nucleus, nucleus of the lateral olfactory tract, periamygdaloid cortex (PAC), also referred to as the posterolateral subdivision of the cortical nucleus by some studies, and posterior subdivision of the cortical nucleus, sometimes called the posteromedial subdivision of the cortical nucleus. (McDonald, 1998, Sah et al., 2003). The cortical nuclei receive inputs from the olfactory bulb, and is implicated in odor driven innate behaviors (Lurilli et al, 2017). The basomedial amygdala, medial amygdala and cortical amygdala’s roles in pain are understudied and should be explored in future studies.

An initial model of nociceptive processing within the amygdala proposed that (1) the LA-BLA complex serves as the primary input station for multimodal sensory and nociceptive information, which is indirectly sent to the CeA, and (2) the CeA functions as the main output station for affect-related regulation of downstream behaviors (reviewed in Neugebauer et al., 2004, Neugebauer et al., 2009). However, this model has been challenged by several studies demonstrating the direct impact of spino-parabrachial input on CeA-mediated output (Liu et al., 2011, Han et al., 2015a, Han et al., 2015b, Torres-Rodriguez et al., 2023).

Furthermore, projections from the BLA/LA coordinating with the prefrontal cortex can also modulate affective-nocifensive behaviors and cognition without CeA involvement (Apkarian et al., 2004, Ji et al., 2010). An earlier study in an inflammatory pain model revealed that a somatic noxious stimulus activated CeA neurons but not BLA neurons, while a visceral noxious stimulus activated both CeA and BLA neurons (Tanimoto et al. 2003). This study demonstrated that both somatic and visceral noxious stimuli in rats produced negative affect as assessed in the conditioned place aversion assay. Notably, the somatic noxious stimuli-induced conditioned place aversion was abolished by either BLA or CeA lesion, whereas the visceral noxious stimuli-induced conditioned place aversion was abolished by CeA lesion but not BLA lesion. This suggests that not all types of nociceptive inputs that influence emotion-affect are dependent on the BLA and the CeA.

To put these inputs and outputs in the correct context, there has been a major drive to use physiology to investigate how defined cell populations and micro-circuits change in the context of nociception and injury. In the remaining sections of this review, we aim to frame the role of the amygdala using these data incorporating classic findings along with newer studies including recent findings using pharmacologic approaches as well as genetically defined cell populations. We analyze the neuronal physiology of each component of the amygdalar complex from a naïve/non-pain condition before describing the pain-related modifications undergone by the different amygdala neuronal types reported.

The flow of nociceptive information from the spinal cord, undergoes complex regulation in various brain regions, of which, the amygdala is an integral part. Nociceptive information transmitted through the thalamo-cortical pathways and spino-parabrachial pathway are regulated within the amygdala to transform these signals into outputs with behavioral and systemic physiological significance. Ultimately, the underlying inherent and plastic amygdala neuronal properties, intra-amygdala fine-tuning, neuronal-type specific inputs, and projection targets define the functional significance of the amygdala.

Most of the LA and BLA consists of glutamatergic excitatory neurons (80–85 %) (Vereczki et al. 2021). While the CeA consists of mostly inhibitory neurons (McDonald, 2003). A recent study reported that all the analyzed CeA neurons robustly clustered to GABA markers. This study incorporated single-cell transcriptomics to map the spatial distribution of amygdala cell types based on their neurotransmitter identity (Hochgerner et al. 2023). The projection targets of CeA divisions are involved in autonomic regulation and state-dependent and scalable action selection functions (Fadok et al. 2018). While the LA-BLA principal neuronal morphology and functional properties resemble that of hippocampal and cortical pyramidal neurons, the CeA neuronal morphology resembles that of striatal neurons (Vasquez et al. 2024).

### 3.1 Neuronal properties of LA-BLA complex under normal conditions

In normal conditions, where the subject or the animal is not injured, LA-BLA neurons respond to somatosensory stimuli including nociceptive stimuli. An *in-vivo* study utilizing extracellular single unit recording from anesthetized adult rats (sex unspecified) demonstrated that BLA neurons exhibited responses to an innocuous stimuli applied to the knee joint and even stronger responses to evoked noxious stimuli (Ji et al. 2010). This study, examining the somatosensory receptive fields of BLA neurons found that they were multi-receptive neurons. That is, these neurons show responses to innocuous stimuli but show stronger responses to noxious stimuli. They also exhibited a tendency to increase their firing rate during both innocuous and noxious stimuli relative to background spike activity. This suggests the presence of neural activity that correspond to somatosensory stimuli within the LA-BLA complex, despite the highly processed nature of the signals received in this nucleus.

The LA-BLA excitatory neurons are typified by earlier studies as being CaMKIIα-positive glutamatergic neurons (McDonald et al. 2002). A recent study in freely behaving mice utilized head-mounted miniature microscopes to visualize the *in-vivo* calcium dynamics in CaMKII-positive BLA neurons (Corder et al. 2019). This study identified a nociceptive ensemble with distinct activity patterns that responded to noxious heat, cold and mechanical stimuli. Larger subsets of this ensemble were activated to increasing intensity of the noxious stimuli. When this nociceptive neuronal ensemble was specifically silenced either chemogenetically or optogenetically, there was a reduction in pain-mediated affective behaviors *in vivo* (Corder et al. 2019).

LA-BLA principal neurons are broadly classified into two types: (1) bursting accommodating type and (2) repetitive firing non-accommodating type (Washburn and Moises, 1992, Rainnie et al., 1993). These studies were performed on brain slices from young adult male rats with voltage measurements made with sharp electrode intracellular recordings. The LA and BLA's repetitive type neurons differ only by the presence of late-firing behavior in the BLA neurons. LA-BLA projection neurons exhibit broad action potentials, and accommodating neurons have a larger afterhyperpolarization (AHP) than repetitively firing neurons (Faber et al. 2001). Nevertheless, pyramidal neuronal firing phenotype based functional role in the context of nociception and pain are yet to be understood.

### 3.2 Interneurons of LA-BLA complex

LA-BLA pyramidal neurons operate under stringent inhibitory control from local interneurons. These interneurons generate typical action potentials with narrower half-widths and exhibit cortical interneuron-like firing properties such as regular spiking, irregular spiking, fast spiking, stuttering, accommodating, and delayed spiking (Sosulina et al., 2010, Spampanato et al., 2011, Polepalli et al., 2020).

Approximately 20 % of the LA-BLA complex consists of interneurons. These cells are non-spiny, their dendrites are smooth, and their axonal collaterals project only locally, functioning as the LA-BLA GABAergic inhibitory microcircuitry (McDonald, 1985, McDonald et al., 2002, Sah et al., 2003). Bienvenu et al. (2012) categorized rat LA-BLA interneuron subtypes from *in to vivo* electrophysiological recordings based on their connectivity patterns, spontaneous firing frequencies, and responses to noxious mechanical stimuli in naïve animals (Bienvenu et al. 2012). This categorization resulted in four classes of LA-BLA interneurons: axo-axonic, CB+ (calbindin-positive) dendrite-targeting, AStria (amygdalo-striatal transition area), and PV+ (parvalbumin-positive).

A recent Immunohistochemical study in mice from both the sexes quantified the ratio of GABAergic neurons and categorized them based on their molecular subtypes identified. The estimates suggest that axo-axonic cells comprise 5.5–6 % of the total, basket cells expressing PV are 17–20 %, those expressing cholecystokinin (CCK) are 7–9 %, somatostatin (SST)-expressing dendrite-targeting inhibitory cells are 10–16 %, NPY-containing neurogliaform cells are 14–15 %, VIP and/or calretinin (CR)-expressing interneuron-selective interneurons are the most abundant at 29–38 %, and finally, GABAergic projection neurons co-expressing SST and neuronal nitric oxide synthase (nNOS) are present at 5.5–8 % (Vereczki et al. 2021).

Detailed reviews on the functional, neurochemical, and anatomical identification of LA-BLA GABAergic interneurons in contexts such as fear conditioning, salience, etc., are available elsewhere (Spampanato et al., 2011, Bienvenu et al., 2012, Capogna, 2014). However, in the context of nociception and pain the functional roles of the different types of LA-BLA local interneurons are yet to understood. Since increased BLA output is considered a contributor to pain-related affective behaviors it is important to explore the role of LA-BLA interneurons in nociception and pain.

### 3.3 Opioid receptors of LA-BLA complex

The three major G-protein-coupled receptors of the endogenous opioid system, the mu-opioid receptor (MOR), delta-opioid receptor (DOR), and kappa-opioid receptor (KOR), are all expressed at high levels in the amygdala, with relative differences between the nuclei. These receptors are implicated in various amygdala-associated functions, including stress adaptive responses and pain modulation (Atweh and Kuhar, 1977, Drolet et al., 2001, Zhang et al., 2015). The involvement of pain related amygdala function is demonstrated in a human study, where naloxone (a MOR antagonist) administration facilitated sustained pain ratings and prevented the attenuation of amygdala activation during pain stimulus-induced conditioned hypoalgesia (Eippert et al. 2008). These data suggest the involvement of the amygdala in opioid-dependent habituation to conditioned stimuli.

Opioid receptor activation mediates intracellular effects such as inhibition of adenyl cyclase, activation of inwardly rectifying potassium currents, and inhibition of voltage-dependent calcium currents (Law et al. 2000). Opioid receptors are expressed in both principal neurons and interneurons of the rodent LA-BLA complex (Sugita and North, 1993, Faber and Sah, 2004, Przybysz et al., 2017). In normal conditions in the LA, MOR activation in brain slices significantly inhibited GABA receptor-mediated inhibitory synaptic potentials by 60 % in unmarked LA neurons. Similarly in LA, DOR activation by DPDPE (Tyr-d-Pen-Gly-Phe-d-Pen), a DOR agonist, reduced presynaptic GABA release, although without postsynaptic effects. In contrast, KOR activation by U50488 (*trans*-3,4-Dichloro-N-methyl-N-[2-(1-pyrrolidinyl)cyclohexyl]-benzeneacetamide), a KOR agonist, had no discernible effect on either inhibitory synaptic inputs or postsynaptic potentials (Sugita and North, 1993). This study was performed on rat brain slices with intracellular recording technique.

MOR activation by DAMGO (D-Ala(2), Nme(4), Gly-ol(5)-enkephalin) hyperpolarized approximately 50 % of neurons, an effect reversed by naloxone and the MOR-selective antagonist CTOP (D-Phe-Cys-Tyr-D-Trp-Orn-Thr-Pen-Thr-NH2) (Sugita and North, 1993). Morphine and DAMGO inhibited spike frequency adaptation in LA pyramidal neurons, decreasing neuronal output through G_i/o_ mechanisms, activating the arachidonic acid pathway, and upregulating voltage-dependent Kv1.1/1.2 potassium currents in rat brain slices (Faber and Sah, 2004). Spike frequency adaptation is a spiking property that increases the threshold for subsequent spikes in an action potential train. It is regulated by ion channels like voltage-gated K+/KCNQ channels.

Under normal conditions, KOR activation inhibited field potentials and long-term potentiation in mouse brain slices (Huge et al. 2009). KOR agonists also increased spontaneous IPSC frequency in adolescent but not in adult rat BLA pyramidal neurons, suggesting age-dependent effects (Przybysz et al. 2017). In contrast, MOR activation decreased presynaptic GABA release onto neurons projecting to the central amygdala, thereby reducing the frequency of miniature IPSCs in 77 % of CeA-projecting BLA neurons. Notably, this effect was mediated through Kv1.1/1.2 potassium channels and could be blocked by dendrotoxins, 4-AP, and tityustoxin-Ka in male rats (Finnegan et al. 2006). Additionally, systemic morphine administration increased spontaneous firing rates in 10 out of 16 BLA neurons in anesthetized cats, an effect that was partially blocked by naloxone, indicating MOR involvement (Chou and Wang, 1977). Further research is needed to fully understand the role of molecularly identified, functionally relevant sub populations of BLA neurons and their opioid receptor expression profiles in potentially producing non-addictive analgesia.

### 3.4 The LA-BLA complex Neurophysiology after injury

In a model of neuropathic pain, *in-vivo* extracellular recording from anesthetized adult male rats showed an increase in background firing and increased evoked firing activity with innocuous and noxious stimuli (Li et al. 2017). The same study showed that a comorbid chronic stress exacerbates the BLA neuronal sensitization. A recent study in an inflammatory pain model in adult male mice showed in brain slices that glutamatergic CamKII BLA neurons exhibited increased firing rates, reduced threshold currents and slight but statistically significant increases in resting membrane potential (Meng et al. 2022). These neurons also exhibited increased mEPSC amplitude and frequency. Interestingly, blocking AMPAR and NMDAR currents resulted in the elimination of pain-induced enhanced membrane excitability measurements such as firing rate, RMP, and threshold current.

In a neuropathic pain model in adult mice, selective inhibition of CaMKII-positive neurons *in-vivo* with DREADD (engineered Designer Receptors Exclusively Activated by Designer Drugs) led to a reduction in mechanical hypersensitivity and alleviated anxiety behavior without affecting overall motor activity This critical involvement of BLA CaMKII-positive neurons was recently shown *in-vivo,* in paclitaxel induced neuropathic pain model in young adult mice (sex unspecified). (Liu et al. 2022). The precise physiological changes, such as firing rate or synaptic transmission, undergone by CaMKII-positive neurons after neuropathic pain is yet to be explored.

Within the context of pain, the BLA and the mPFC are important interconnected brain regions, specifically, the prelimbic mPFC-to-BLA projections are implicated in regulation of pain-mediated stress in adult mice of both sexes (Little and Carter, 2013, Liu et al., 2020). The PFC nuclei are involved in higher cognitive functions such as decision making and goal-directed behaviors. Painful stimuli can impair these higher cognitive functions in male and female human subjects (Apkarian et al. 2004). Studies from an animal model suggest that injury-induced BLA activation can drive such impairment. For instance, BLA-driven mPFC inactivation induced by injury leads to cognitive impairment in an arthritic pain model in anesthetized adult rats (Ji et al. 2010). This study also showed increased firing frequency after pain induction *in-vivo*, and increased firing frequency and EPSC amplitude in the BLA from brain slices from arthritic rats. This pain-related BLA neuronal activation and the subsequent mPFC inactivation were reversed when a corticotropin-releasing factor 1 receptor (CRF1) antagonist was introduced into the BLA *in-vivo.* This reversal was accompanied by behavioral responses such as reversal of pain-related nocifensive behavior and decision-making deficits. In brain slices from arthritic rats, introducing the CRF1 receptor antagonist NBI27914 into the BLA inhibited synaptic plasticity, observed as reduced miniature and evoked EPSCs. Notably, this appears to be a CeA-independent process, suggesting a separate amygdala circuitry in the cognitive-emotional dimension of pain-processing (Ji et al. 2010). The LA-BLA neuronal changes observed here are in cells that are not specific to any molecularly identified subtypes. Table 1 summarizes the effect of different pain models on BLA neurons.

**Table 1:** Summary of the pain models and the modification undergone by LA-BLA neurons.

***Lateral − Basolateral Amygdala***							
**Pain Model**	**Species**	**Sex**	**Age**	**Injury-Type**	**Modification**	**Side**	**Reference**
Neuropathic Pain	Rat (Sprague Dawley) *In-vivo*, anetheti-zed	Male	150–180 g	Spared Nerve Injury	Increased background and evoked firing.	Right	Li et al., 2017
	Rat (Sprague Dawley) *Ex-vivo*	Male	150–180 g	Spared Nerve Injury	In Unmarked cells. Enhanced evoked spiking activity in comorbid chronic stress.	Right	Li et al., 2017
Inflammatory Pain	Mouse (C57BL/6) *Ex-vivo*	Male	8–12 wk	Complete Freund's Adjuvant	In CamKII neurons. Enhanced excitatory transmission (AMPAR and NMDAR) mediated increase in firing rate.	Not specified	Meng et al, 2022
Arthritis Pain	Rat (Sprague Dawley) *In-vivo*, anetheti-zed	Male	250–350 g	Kaolin/ Carrageenaninto left knee	Increased background, innocuous and noxious evoked spiking response.	Right	Ji et al, 2010
	Rat (Sprague Dawley) *Ex-vivo*	Male	250–350 g	Kaolin/ Carrageenaninto left knee	In Unmarked cells. Increased evoked EPSC amplitude.	Right	Ji et al, 2010

Single-cell RNA sequencing in the mouse BLA has identified two discretely separated glutamatergic populations based on clusters of differentially enriched genes (O’Leary et al. 2020). While this study elucidates the heterogeneity of the BLA population, further research is needed to identify functionally relevant non-overlapping populations in the context of nociception, showing distinct and well-separated transcriptome differences. Kim et al. (2016) conducted an activity-dependent transcriptional profiling of BLA neuronal populations from adult male mice activated during negative and positive stimuli, where two non-overlapping populations showed enriched expression of *Rspo2* and *Ppp1r1b,* respectively*.* These potential markers for negative and positive valence encoding neurons were further corroborated by Corder et al. (2019) in adult male mice. The “negative” stimuli given here was foot shock, while the “positive” stimuli was exposure to a female mouse. As a site of associative learning, the LA-BLA encodes neutral conditioned stimuli that are associated with the valence of the unconditioned stimuli (Zhang et al., 2013, Beyeler et al., 2018). Therefore, it is important to study the physiology of the negative-valence encoding cell-types involved in different pain/injury models.

For the BLA, pain-related plasticity mechanisms in cell-type specific studies have not been extensively explored but should be a major focus of future studies. This will disentangle the role of these heterogeneous cells in nocifensive behavior versus other behavioral outputs including learning and memory. Tools that could be used in these studies include Cre driver lines for the valence encoding excitatory BLA neuronal population, Crtpt-Cre line (for *Ppp1r1b^+^*) and Rspo2-Cre line (for *Rspo2^+^*), and inhibitory BLA neurons, CCK-Cre (Cholecystokinin^+^), (Kim et al. 2016).

### 4.1 CeA complex under normal conditions

Within the CeA, the latero-capsular (CeC) region has been identified for its reception of different types of nociceptive synaptic inputs from the PBN (Bernard et al. 1993) and polymodal inputs from the BLA (Sah et al. 2003). Earlier studies from extracellular recordings of anesthetized rats estimated that the majority of the CeC and CeL neurons (about 80 %) respond to noxious stimuli (Bernard et al. 1992). These rats were anesthetized with 2 % halothane in a nitrous oxide-oxygen mixture along with a nerve impulse blocker (Gallamine triethiodide) and a muscle relaxant (Atropine sulfate). Of the total population, the neurons that exhibited excitatory responses (increased firing activity) to noxious stimuli (mechanical pinch and noxious thermal stimuli) were about 46 % and the neurons that exhibited inhibitory responses (reduced spontaneous firing activity) comprised 34 % of the total. Of the CeA population that were excited during noxious stimuli, 77 % responded with high magnitude, of which 75 % responded exclusively to noxious stimuli and 25 % responded preferentially to the noxious stimuli. Of the CeA neuronal population that were inhibited by noxious stimuli, 60 % showed more than a 50 % reduction in spontaneous firing during noxious stimulation. Of this prominently inhibited population, 81 % exclusively responded to noxious stimuli. The other 19 % responded preferentially to noxious stimuli but also responded to a lesser degree to innocuous mechanical stimuli such as slight pressure, brushing or touch. Notably, in that study the neurons were not identified molecularly so it is not clear the mechanisms of the observed heterogeneity.

The CeC, CeL, and CeM of the central amygdala receive glutamatergic inputs that include both AMPA and NMDA receptor-mediated excitatory transmission (Sah and De Armentia, 2006). In addition, glutamate-activated mGluRs are implicated in both excitatory and inhibitory CeA synaptic transmission in pain related plasticity in adult male rats (Han et al., 2006, Ren and Neugebauer, 2010). Under normal conditions, when mGluR1 (group I mGluR) is antagonized pharmacologically, no change in basal synaptic transmission levels is observed, however, when mGluR5 (group I mGluR) is inhibited, both inhibitory and excitatory transmission is reduced (Ren and Neugebauer, 2010).

CeA neurons’ inhibitory synaptic transmission in rats is through GABA_A_ receptors and densely express GABA_C_ receptors, which are negatively modulated by benzodiazepines (Delaney and Sah, 1999). Within the divisions of the CeA, there are three major distinct classes of neuronal populations as defined by spiking patterns in adult male rats and mice – 1) Early spiking (ES) cells, 2) late spiking (LS) cells and 3) low-threshold bursting neurons (Chieng et al., 2006, Haubensak et al., 2010, Adke et al., 2021). In mice, within the CeL, the early spiking and the late-spiking cells form distinct autaptic connections, where ES neurons exhibit higher degree of autaptic regulation of spike timing than the LS neurons (Hou et al. 2016). Autapses are typical of GABAergic interneurons where the axons of a neuron innervate its own dendrite. Such an architecture enhances spike-timing precision where GABAergic transmission evoked by a neuron’s action potential results in hyperpolarization, thus regulating the timing of the next spike. 95 % of CeM cells are late-firing neurons and display a marked voltage and time-dependent outward rectification in the depolarizing direction (Martina et al. 1999). In contrast, only 56 % of the CeL cells are late-firing type. The CeL population that lacked the late-firing characteristics showed inward rectification in the hyperpolarizing direction. These studies were done on brain slices from guinea pigs from either sex.

CeA neurons have been known to project to periaqueductal gray (PAG), a midbrain structure that is involved in analgesia and other functions in rats. CeA and PAG also reciprocally connect (Rizvi et al. 1991). These reciprocal connections are critical in nociceptive processing because of the location of the PAG nuclei. While the PAG is part of ascending nociceptive pathway, it is also a major region of descending pain inhibitory pathway (Behbehani, 1995). Recent studies have been exploring the projection-specific physiological properties of CeA neurons in mice. A notable *ex-vivo* study from both male and female mice performed detailed physiological characterization of the CeA neurons projecting to PAG (CeA-PAG) (Li and Sheets, 2018). CeM-to-PAG projecting neurons (CeM-PAG) had distinct subthreshold properties such as resting potential, voltage sag and input resistance compared to CeL-to-PAG projecting neurons (CeL-PAG). Also, CeM-PAG neurons exhibited distinct firing properties assessed by voltage threshold, current threshold, action potential onset, firing frequency, half-width, height and spike frequency adaptation. Further, a majority of the CeL-PAG neurons were late-firing while the CeM-PAG neurons consisted of a heterogenous firing phenotypes that includes regular spiking (25 %), fast-spiking (34 %), bursting (31 %) and late-firing (9 %) types. These characterizations, however, are not based on molecular identities. The study further explored pain induced modifications in the intrinsic properties of CeA-PAG neurons in a model of inflammatory pain. The results are discussed in section 4.3.

Several recent RNA transcriptomic studies from mice have categorized CeA neurons into more than 20 subtypes which include cells expressing protein kinase c-delta (*prkcd*), somatostatin (*sst)*, corticotropin releasing factor *(crf),* cocaine and amphetamine regulated transcript *(cartpt),* tachykinin precursor 1 *(tac1),* sodium channel subunit beta-4 *(scn4b),* vitamin D (1,25- dihydroxyvitamin D3) receptor *(vdr),* and nuclear receptor subfamily 2 group f member 2 *(nr2f2)* (O’Leary et al., 2022, Wang et al., 2023). The major genetically marked neuronal subtypes of the CeC that have been intensely studied are those that express protein kinase C delta (PKCδ), somatostatin (SST), calcitonin gene related peptide (CGRP) receptor (aka calcitonin receptor-like receptor (*calcrl*)), neurotensin (Nts) and corticotropin releasing factor (CRF). A detailed account of the co-expression patterns of these cell-type markers within the mice CeA sub-nuclei and across the anterior-posterior axis has been published (McCullough et al. 2018). The CeC neuronal population in mice contains heterogenous firing phenotypes with ES and LS and low threshold firing neurons (Adke et al. 2021). Recent brain slices electrophysiological studies have made similar observations in genetically marked populations such as PKCδ and SST where their firing properties were heterogenous and there were marked differences in neuronal excitability (Adke et al. 2021).

Electrophysiological properties of SST+ cells within the CeL have been characterized in mice brain slice of either sex in normal conditions (Mork et al. 2022). This study found that 56.9 % of the CeL SST neurons were early-spiking type, 43 % were late-firing type, and about 5 % were spontaneously firing neurons. Compared to late-firing neurons, the early spiking neurons needed a smaller current to trigger APs, and were more depolarized, had a narrower action potential half-width, and showed less spike frequency adaptation. In rats, CRF neurons which are located mainly in the CeL, showed a late firing pattern with a low incidence of regular spiking and low threshold bursting types *ex-vivo* (Pomrenze et al., 2015, Kiritoshi et al., 2024).

The CeC receives nociceptive inputs from CGRP-expressing lateral PBN neurons, which are the main, likely only, source of CGRP in the amygdala (Schwaber et al., 1988, Kruger et al., 1988, Gauriau and Bernard, 2002, Dobolyi et al., 2005). Many studies have pointed out the critical role of CGRP in facilitating CeA plasticity in normal conditions and after injury. *Ex-vivo* studies from uninjured mice and rats showed that when CGRP is exogenously introduced on brain slices, an enhanced NMDAR-mediated excitatory current dependent on protein kinase-A (PKA) is observed (Han et al., 2010, Okutsu et al., 2017). However, the CGRP-mediated facilitatory effect on the excitatory post-synaptic currents (EPSCs) did not affect the baseline AMPAR-mediated EPSCs (Okutsu et al. 2017). Also, CGRP on mice (Allen et al. 2023) and rat (Han et al. 2010) brain slices produced increased firing frequency in *calcrl*^+^ cells and unmarked cells, respectively.

The firing phenotypes of CRF neurons were described in earlier studies as regular spikers and low threshold bursting cell types (Herman et al., 2013, Herman et al., 2016). When CRF is super fused on brain slices from uninjured adult male rats, the neurons recorded from CeC and CeL showed synaptic facilitation by increased EPSCs (NMDA-mediated) dependent upon CRF1 receptor and PKA (Ji et al. 2013). In the same study, micro-dialysis of CRF *in-vivo* into the CeA triggered pain-like behavioral responses such as increased vocalizations and spinal responses (Ji et al. 2013). After super fusing the peptide CRF in the brain slices from normal rats, an enhanced firing rate and enhanced mEPSC was recorded in unmarked neurons from CeC and CeL (Ji et al. 2013). CRF application did not affect the frequency of mEPSC, suggesting that the mEPSC enhancement is post-synaptic. Of the known CeA cell-types, CRF neurons are well studied for their role in stress (Bruchas et al., 2010, Crowley et al., 2016), especially the role of CRF neurons co-expressing dynorphin/kappa opioid receptors (Marchant et al., 2007, Pomrenze et al., 2015). Optogenetic activation of CRF neurons induced pain and anxiety like behavior in non-injured rats (Mazzitelli et al. 2022). Superfusing CRF onto CeA containing brain slices from wildtype mice and CRF2 knock out mice showed enhanced GABAergic synaptic transmission measured as increased GABA_A_ mediated IPSC amplitude (Nie et al. 2004) and frequency (Roberto et al. 2010).

Some of the afferent projections received by the CeA from the PBN were recently delineated in non-injured adult male and female mice. This *ex-vivo* study found that PBN projections that make monosynaptic connections to CeA subdivisions are organized topographically in a cell-type specific manner (Li and Sheets, 2020). The study employed channel-rhodopsin expression system in PBN to test the monosynaptic connections by evoking the PBN axonal terminals at CeA subdivisions. In the CeC, SST− neurons exhibited significantly larger monosynaptic EPSC amplitudes compared to SST+ neurons, while PPR (paired-pulse ratio) remained similar between the two groups. In the CeL, no significant differences were observed in the amplitude or PPR of evoked EPSCs between SST+ and SST− neurons. In the CeM, SST+ neurons showed significantly larger EPSC amplitudes than SST− neurons, despite no significant differences in PPR. Overall, these findings suggest that the efficiency of presynaptic glutamate release from PBN inputs does not account for the amplitude differences seen between SST+ and SST− neurons in the CeL and CeM. Furthermore, the consistent observation of a smaller EPSC amplitude following the second light pulse in all PPR recordings indicates a high release probability for PBN synapses targeting CeA neurons.

Similarly, in corticotropin-releasing hormone (CRH)-tdTomato mice (synonymous with CRF neurons), evoking PBN axon terminals elicited monosynaptic EPSCs in subsets of both CRH+ and CRH− neurons within the CeL and CeM. CRH+ neurons were not found in the CeC. In both the CeL and CeM, no significant differences were observed in either EPSC amplitude or PPR when comparing CRH+ neurons to CRH− neurons. Notably, only a portion of both CRH+ and CRH− neurons in the CeM exhibited EPSCs following PBN input stimulation. These results suggest that similar to the SST+ and SST− findings, the functional organization of PBN inputs to the CeA does not reveal a significant difference in synaptic strength or release probability between CRH+ and CRH− neurons.

### 4.2 Opioid receptors of CeA

Several studies have been conducted that showed the effects of different opioid receptor agonists on the physiology of CeA neurons in normal conditions. Introducing a DOR agonist DPDPE on mice brain slices decreased mIPSC frequency in 60 % of CeM cells (Kang-Park et al. 2007). However, the DOR agonist had no effect on eEPSCs or eIPSCs on brain slices from male rats (Zhu and Pan, 2005, Bie et al., 2009), suggesting that DOR activation-mediated inhibition is through a reduction in presynaptic GABA release in CeM. Activation of KOR induced a similar inhibitory effect on brain slices from adult male rats, when endogenous peptide (Dynorphin − DYN[1–17]) or U69595, decreased evoked IPSP amplitude in approximately 80 % of cells in the CeM (Gilpin et al. 2014), and a highly selective KOR agonist U69593 on brain slices from male mice decreased spontaneous IPSC frequency (Kang-Park et al. 2013). This suggests that activating KOR induces a reduction in the inhibitory synaptic transmission at the pre- and post-synapses in the CeM (Przybysz et al. 2017). KORs are also implicated in inhibition of tonic GABA release (Gilpin et al. 2014). Similar to DOR and KOR, activating MOR produced reduction in inhibitory synaptic transmission. The MOR agonist DAMGO introduced on male mice brain slices decreased eIPSCs from BLA stimulation (Blaesse et al. 2015). DAMGO also decreased mIPSC frequency and decreased the eIPSC amplitudes in CeA neurons projecting to the ventrolateral periaqueductal gray, indicating reduced presynaptic GABA release from brain slices in male rats (Finnegan et al. 2005). MOR agonist (Met-ENK and DAMGO) also reduced evoked EPSC amplitudes following stimulation in the CeM or BLA in rat brain slices. Met-ENK also increased paired pulse ratio and decreased the frequency of mEPSCs, suggesting a decrease in presynaptic glutamate release. MOR’s presynaptic effect involves 4-aminopyridine sensitive potassium channels (Zhu and Pan, 2005).

Other than modulating synaptic transmission, opioid receptor activation produced hyperpolarizing membrane currents. Activating DOR on rat brain slices by introducing deltorphin II (DOR agonist) induced an outward potassium current in approximately 18 % of low threshold bursting neurons in the CeM (Chieng et al. 2006). Activation of DOR1 in the CeA inhibits both anxiolytic BLA and anxiogenic PBN excitatory inputs. In a model of inflammatory pain (Complete Freund’s Adjuvant), DOR2 activation doesn't affect CeA excitatory transmission in normal and 4-hour CFA mice but inhibits PBN input in 7-day CFA mice. This effect was not seen at the BLA-CeA synapse. By day 21 post-CFA, both DOR1 and DOR2 function in the CeA is undetectable. This suggests that the roles of DOR1 and DOR2 in modulating specific BLA-CeA and PBN-CeA pathways shift across different stages of pain-related anxiety (Zhou et al. 2021). This can be advantageous for potential treatment for pain and anxiety with DOR agonists. Similar to DOR activation, KOR agonist U69593 on adult male rat brain slices also induced an outward potassium current in 17 % of CeA neurons (including CeC, CeL and CeM) (Chieng et al. 2006). Another study found that the KOR agonist U69593 induced an outward current in approximately 50 % of accommodating type CeA neurons, but not in non-accommodating type CeA neurons, indicating postsynaptic inhibition in a specific subset of CeA neurons in brain slices of male rats (Zhu and Pan, 2004). Activation of MOR with DAMGO and Met-ENK produced a similar effect where the agonist induced a G-protein-coupled inward rectifying potassium (GIRK) channel-mediated outward current in the non-accommodating type CeL and CeM neurons in rat brain slices (Zhu and Pan, 2004). Administering morphine *in-vivo* in anesthetized male rats decreased glutamate-driven firing rate in more than 75 % of CeA neurons, indicating inhibition of glutamate-induced firing rate (Freedman and Aghajanian, 1985). Overall, 61 % of CeC, CeL and CeM neurons responded to DAMGO by inducing the outward potassium currents, indicating high MOR expression across CeA sub-divisions (Chieng et al. 2006).

A recent study showed that KOR activation mediated disinhibition in the CRF neurons induce anxiety-like behaviors and avoidance behavior in male rats (Hein et al. 2021). Administration of U-69593 (KOR agonist) directly into the CeA of naïve adult male rats *in-vivo* resulted in increased vocalizations to noxious mechanical stimuli, induced anxiety-like behaviors in the open-field test and exhibited avoidance in the conditioned place preference test. These KOR agonist-mediated behavioral outputs were attenuated when CRF neurons were silenced optogenetically. The agonist’s action *ex-vivo* reduced the frequency of inhibitory currents without any change in either the EPSC or IPSC amplitude. The study also observed that the PBN-evoked feed-forward inhibition on CRF neurons was reduced by the KOR agonist. Since the opioid receptor expression is spread across CeA subdivisions, a cell-type specific understanding of the physiology and the functional output of the opioid manipulations on those distinct cell-types under normal and pain-conditions are necessary.

### 4.3 CeA neuronal physiology after injury using pharmacologic approaches

Previous reviews that have discussed amygdala in the context of pain, focused on anatomical, morphological and circuitry aspects (see Veinante et al., 2013, Neugebauer, 2020). In this section 4.3, we briefly describe physiology papers that focus on pharmacology and physiology to explore pain related changes in unmarked CeA neurons.

An earlier study, in a model of arthritis pain, examined CeA neuronal responses in persistent pain in rats (Neugebauer et al. 2003). Extracellular recordings were performed *in-vivo* on anesthetized adult male rats (pentobarbital sodium) while mechanical and thermal stimuli were given, before and after inducing knee arthritis. Based on the responses neurons were categorized into 1) multi-receptive MR) neurons, which respond to various stimuli, showed enhanced responses to mechanical stimuli and expanded receptive fields after arthritis. These changes occurred in two phases. Thermal responses in these neurons remained unchanged, but background and electrically evoked activity increased, 2) Nociceptive-specific (NS) neurons, which specifically respond to painful stimuli, showed no response changes after arthritis, and 3) Somatosensory (somesthetic), a third group of neurons, initially unresponsive to somatosensory stimuli, developed prolonged mechanical responses in the arthritic state. The study concluded that prolonged pain enhances the responsiveness of a subset of CeA neurons, particularly to mechanical stimuli, suggesting a specific sensitization rather than general hyperexcitability. MR neurons may integrate pain-related information, and the recruitment of previously unresponsive neurons increases amygdala processing. NS neurons maintain the distinction between painful and non-painful inputs.

Further studies showed input specific synaptic plasticity at the CeA. Enhanced excitatory synaptic transmission at the PBN projections to the CeA (PBN-CeC) has been observed in various pain models (Ikeda et al., 2007, Cheng et al., 2011). Interestingly, different pain models seem to follow diverse routes in potentiating pain-mediated excitatory synaptic transmission within the CeC. In an arthritis pain model, at the PBN-CeC synapses, NMDA and non-NMDA mediated ionotropic glutamatergic transmission is necessary for CeC neurons’ hyperactivity in unidentified post-synaptic CeA neurons (Li and Neugebauer, 2004). Adult male rats under anesthesia (pentobarbital sodium) were used to record extracellular single neuron activity. In the same arthritis model, it was observed that the synaptic plasticity was associated with increased mGluR1 pre-synaptic function. The expression levels of both mGluR1 and mGluR5 were upregulated in the CeA after arthritic injury (Neugebauer et al. 2003). Functionally, in the post-synaptic CeC neurons from male rat brain slices, the input–output functions of excitatory transmission increased while the inhibitory transmission decreased suggesting a pain-related plasticity mechanism in feedforward inhibitory control. This pain-related effect in the synaptic transmission was reversed when mGluR1 (group I mGluR) was pharmacologically inhibited (Ren and Neugebauer, 2010). This suggests mGluR1′s roles as a pain-mediated synaptic plasticity modulator through a disinhibition mechanism. In the same arthritis model, similar to normal conditions, inhibiting mGluR5 with an antagonist reduced both inhibitory and excitatory transmission (Ren and Neugebauer, 2010). Conversely, in a model of arthritis, applying a group II mGluR agonist (LY354740) on rat brain slices produced stronger reductions in the evoked EPSC amplitude at PBN-CeA synapses in CeC and CeL neurons. However, the agonist action on miniature EPSCs recordings showed a reduction in frequency but not amplitude, suggesting the involvement of a presynaptic mechanism in arthritis pain. The inhibitory effect of the agonist did not affect GABA transmission (Han et al. 2006).

In a model of neuropathic pain (spared nerve injury), *in-vivo* extracellular recordings from adult male rats showed increased background spiking with innocuous stimuli and noxious stimuli evoking spiking activity (Li et al. 2017). The same study showed an enhanced LTP (long-term potentiation) at the BLA-CeA synapse in brain slices after pre-exposure of spared nerve injury (SNI) rats to chronic stress. Interestingly, in another model of neuropathic pain (spinal nerve ligation), an enhanced LTD (long-term depression) was observed at the BLA-CeA synapse in male rat brain slices (Jiang et al. 2021). Both these studies show that BLA-CeA synapses of unmarked CeA cells undergo plasticity that includes LTP and LTP. This observation raises the possibility that different subsets of CeA neuron follow different forms of plasticity and/or different pain models follow different routes of plasticity.

In section 4.1, a recent study (Li and Sheets, 2018) describing CeA-PAG projecting neurons and their distinct CeA nuclei specific intrinsic properties were briefly discussed. The study recorded the intrinsic neuronal property changes in mice brain slices after an inflammatory insult. One day post peripheral CFA induced inflammation, CeL-PAG neuronal excitability remained unaltered. But within the CeM-PAG projections’ only fast spiking subtype exhibited increased excitability 1 day post CFA injection.

These results further strengthen the need to continue to characterize neurons based on their physiology, projection target in the context of pain. CeM being a major output nucleus of the amygdala along with CeC and CeL, their projection specific physiology in the context of nociception and pain is poorly understood.

### 4.4 CeA specific neuronal physiology after injury using Genetic approaches

Recent studies have utilized molecularly identified cell types to perform targeted electrophysiological experiments. These models primarily rely on promotor-based Cre-recombinase driver lines. The advantages of these systems are conditional expression of the reporter genes on specific neuronal populations along with the selective activation or inactivation of neuronal cell-types. Although refined strategies are implemented to insert Cre-recombinase into the gene of interest, the disadvantage of many lines is that there can be a transient expression of Cre-recombinase in the germline or during an earlier developmental stage. This leads to an undesired effect of non-specific expression of Cre-recombinase and the reporter gene across several cell-types and organs. In other words, the fidelity of Cre-recombinase reporter lines should not be assumed to be high. To monitor unwanted expression patterns in the offspring of the Cre-driver line and reporter line, the expression patterns of the reporter gene and the gene of interest under whose promoter Cre recombinase is expressed must be carefully documented using immunohistochemistry and/or *in situ* hybridization. One such expression pattern is seen in the offspring of SST-cre driver and td-Tomato responder line (Mork et al. 2022). This study documented that only 50 % of td-Tomato expressing cells express SST mRNA in the CeA of adult mice. Thus, the interpretations from studies utilizing this Cre-driver line must be considered with caution.

In a neuropathic model of pain, from brain slices of adult male mice, in the CeC, PKCδ and SST neurons seem to have opposing functions, where PKCδ cells showed increased activity while SST neurons showed decreased excitability (Wilson et al. 2019). In these studies, the fluorescent reporter, tdTomato, is expressed under control of the PKCδ or SST promoter through indirect expression of Cre-recombinase. The nerve-injury related increase in PKCδ excitability was observed only in the late-firing neurons with increased input resistance, increased current-injected firing rate and decreased rheobase (Wilson et al. 2019). Also, the proportion of LF, RS and spontaneously firing neurons remained unchanged in pain condition. In SST neurons, pain related reduction in neuronal excitability was observed as a reduction in the proportion of spontaneously active cells (Wilson et al. 2019). The nature of pre-synaptic excitatory transmission at the PBN-CeA SST neurons of the CeC altered after nerve injury (Li and Sheets, 2020). This alteration is observed as an enhancement in the optogenetically evoked paired-pulse ratio after SNI in SST cells *ex-vivo* in mice.

Corroborating with the observation that PKCδ cells showed pain-related increase in excitability, when PKCδ cells were specifically inhibited chemogenetically in the CeA, a reversal of the neuropathic-injury induced mechanical hypersensitivity was observed. Interestingly, a study reported that an ensemble of CeA neurons recorded *in-vivo* in adult male and female mice were activated by general anesthesia (Isoflurane or ketamine/xylazine), with a substantial subset (79.2 ± 12.6 %) of this population overlapping with PKCδ cells. Selective activation of this general anesthesia ensemble reduced nocifensive reflexes across sensory modalities (Hua et al. 2020). It is important to note that usually PKCδ neurons of the CeC are pro-nociceptive (Cui et al., 2017, Wilson et al., 2019), yet, the general anesthesia activated PKCδ population, found localized more in the posterior CeC, were anti-nociceptive. This suggests that different subsets of PKCδ populations within the CeC drive pro-nociceptive and anti-nociceptive responses. What is unknown is the physiological responses of those anti-nociceptive PKCδ neurons since only calcium imaging was used in a non-cell type specific fashion to probe general anesthesia excitability (Hua et al. 2020). Moreover, a recent study showed an anatomical distribution pattern of a subset of PKCδ neurons that overlap with *calcrl*^+^ neurons, where the differences in distribution were evident along the anterior-posterior axis in the CeA (Bowen et al. 2023).

Contrary to PKCδ cells’ excitability post-neuropathic injury in the CeA, SST neurons on the other hand, showed a reduction in excitability. When SST neurons are activated in the CeA, in injured mice, a reversal in neuropathic-injury induced hypersensitivity neuropathic-injury was observed (Wilson et al. 2019). A more recent study has indicated that a subset of SST neurons of the regular spiking type can exhibit enhanced excitability at the chronic phase post-neuropathic pain (Kiritoshi et al. 2024). This suggests that a subset of SST neurons with a specific firing property are pro-nociceptive during the chronic phase of neuropathic injury-induced pain in mice. In a model of neuropathic pain (SNI) in mice of either sex, a differential alteration of PPR of PBN input across CeA subregions was observed on brain slices (Li and Sheets, 2020). Specifically, SNI significantly increased the PPR of PBN input to SST+ neurons in the CeC, while conversely, it significantly decreased the PPR of PBN input to SST− neurons in the CeC. In the CeL, SNI did not alter the PPR of PBN input to either SST+ or SST− neurons. Similarly, in the CeM, SNI did not change the PPR of PBN input to SST+ neurons, but it did significantly increase the PPR of PBN input to SST− neurons. These results suggest that SNI has a region-specific and cell-type-specific effect on the synaptic efficacy of PBN input to CeA neurons, attenuating it in CeC-SST+ and CeM-SST− neurons, and increasing it in CeC-SST− neurons.

A recent study in brain slices from adult male rats using a CRF-Cre x AAV-mCherry reporter evaluated CRF neurons after spinal nerve ligation (SNL) model of neuropathic pain. During the acute phase post injury, CRF neurons at CeC and CeL exhibited an enhanced neuronal excitability and an increase in excitatory synaptic transmission when evoked at presynaptic PBN terminals (Kiritoshi et al. 2024). Although the CRF neurons receiving PBN inputs did not continue to exhibit this trend through the chronic phase (27 days post-injury), interestingly, these animals showed mechanical hypersensitivity both at acute and chronic phase post neuropathic injury. However, an earlier study showed that optogenetic silencing of CeA’s CRF neurons decreased emotional affective responses in acute and chronic phases of the same neuropathic pain model (SNL) (Mazzitelli et al. 2022), suggesting that at the chronic phase of injury, the CRF neuronal excitability seems to be not necessary for the maintenance of injury associated mechanical hypersensitivity and emotional affective behaviors warranting an alternate explanation.

In a model of neuropathic pain (SNI), an ex-vivo study from adult male and female mice expressing tdTomato from a CRH-IRES-Cre transgene showed that changes in PBN input to CRH neurons varied across subregions and cell types. SNI increased PPR in both CRH+ and CRH− neurons within the CeL. In the CeM, SNI decreased PPR specifically in CRH+ neurons, while having no effect on CRH− neurons. These findings demonstrate that SNI induces region- and cell-type-specific alterations in the synaptic strength of PBN inputs to CeA neurons (Li and Sheets, 2020). Although using pharmacological approaches, there is indirect evidence showing the role of CGRPR neurons in pain associated enhancement in synaptic transmission, a comprehensive characterization of CGRPR neurons in a pain model is yet to be performed. The only exception is, in a mouse model of neuropathic pain, in the chronic phase of injury, CGRPR neurons did not show any injury associated modification in excitability (Kiritoshi et al, 2024). The firing properties at the acute phase is yet to be explored along with any possible injury associated modifications in synaptic transmission at both acute and chronic phase of injury. A comprehensive summary of the pain models and the modification undergone by CeA neurons are given in Table 2.

**Table 2:** **Summary of pain models and the modification undergone by CeA neurons.** BLA − Basolateral Amygdala, CeA − Central Amygdala, CeC − Capsular CeA, CeL − Lateral CeA, CeM − Medial CeA, CRH − Corticotropin Releasing Hormone, GluN2B − Glutamate Receptor Subunit of NMDAR, LA − Lateral Amygdala, LTD − Long Term Depression, LTP − Long Term Potentiation, NMDAR − N-methyl-D-aspartate Receptor, PBN − Parabrachial Nuclei, SST – Somatostatin, PKCδ – Protein Kinase C delta.

***Central Nucleus of the Amygdala***								
**Pain Model**	**Species**	**Sex**	**Age**	**Division**	**Injury-Type**	**Modification**	**Side**	**Reference**
Neuropathic Pain	Rat (Wistar) *Ex-vivo*	Both	20–26 Days old	Not specified	Spinal NerveLigation	In unmarked cells. Enhanced NMDAR independent glutamatergic transmission at PBN-CeA synapse.	Right	Ikeda et al. 2007
	Rat (Sprague Dawley) *Ex-vivo*	Male	150–200 g	Not specified	Spinal NerveLigation	In unmarked cells. Enhanced LTD at LA/BLA-CeA synapse in comorbid depressive like syndromes. AMPAR involved.	Not specified	Jiang et al. 2021
	Rat (Sprague Dawley) *Ex-vivo*	Male	150–180 g	Not specified	Spared Nerve Injury	In unmarked cells. Enhanced LTP in comorbid chronic stress. NMDAR (GluN2B) involved at PBN-CeA synapse.	Right	Li et al. 2017
	Rat (Sprague Dawley) *In-vivo*, anethetized	Male	150–180 g	Not specified	Spared Nerve Injury	In unmarked cells. Increased background and evoked firing. Enhanced LTP in comorbid chronic stress. NMDAR (GluN2B) involved at PBN-CeA synapse.	Right	Li et al. 2017
	Mouse PKCδ-Cre (C57BL/6) *Ex-vivo*	Male	8–17 wk old	CeL/CeC	Sciatic cuff implantation	PKCδ, late-firing type. Increased firing rate.	Right	Wilson et al. 2019
	Mouse SST-Cre (C57BL/6) *Ex-vivo*	Male	8–17 wk old	CeL/CeC	Sciatic cuff implantation	SST, spontaneous firing type. Reduction in proportion of spontaneous firing type.	Right	Wilson et al. 2019
	Mouse SST-Cre (C57BL/6) *Ex-vivo*	Both	9–10 wk old	CeC	Sciatic cuff implantation	SST, Regular firing type. Increased firing at chronic pain phase.	Right	Kiritoshi et al. 2024
	Mouse SST-Cre (C57BL/6) *Ex-vivo*	Both	Not specified	CeC	Spared Nerve Injury	SST, unspecified firing type. Increased paired-pulse ratio at PBN-CeC synapse.	Right	Li and Sheets, 2020
	Rat Crh-Cre *Ex-vivo*	Male	4 wk old	Cel/CeC	Spinal NerveLigation	CRH neurons. Increased firing rate at acute phase. Increased excitatory transmission at PBN-CeC/CeL synapse.	Right	Kiritoshi et al. 2024
	Mouse Crh-Cre (C57BL/6) *Ex-vivo*	Both	Not specified	CeL	Spared Nerve Injury	CRH neurons. Increased paired-pulse ratio at PBN-CeL synapse.	Right	Li and Sheets, 2020
	Mouse Crh-Cre (C57BL/6) *Ex-vivo*	Both	Not specified	CeM	Spared Nerve Injury	CRH neurons. Decreased paired-pulse ratio at PBN-CeM synapse.	Right	Li and Sheets, 2020
Arthritis Pain	Rat (Sprague Dawley) *Ex-vivo*	Not specified	120–400 g	CeC	Kaolin/ Carrageenaninto left knee	In unmarked cells. ERK, PKA and CGRPR1 involved increase in EPSCs. NMDAR involved.	Not specified	Han et al., 2005, Bird et al., 2005, Fu et al., 2008
	Rat (Sprague Dawley) *In-vivo*, anethetized	Male	220–400 g	CeC	Kaolin/ Carrageenaninto left knee	In unmarked cells. Increased background, innocuous and noxious evoked spiking response. NMDAR & non-NMDAR involved.	Right	Neugebauer and Li, 2003, Li and Neugebauer, 2004
	Rat (Sprague Dawley) *Ex-vivo*	Male	120–250 g	CeC	Kaolin/ Carrageenaninto left knee	In unmarked cells. Increased BLA-CeC excitatory transmission. Reduced ITC-CeA feed forward inhibition.	Right	Ren and Neugebauer, 2010
Inflammatory Pain	Mouse (C57BL/6) *Ex-vivo*	Male	17–25 Days old	CeC	Formalin Intra-plantar hind paw injection	In unmarked cells. Increased spiking,increased eEPSCs.	Right	Adedoyin et al. 2010
	RatWistar	Male	7–9 wk old	CeC	Formalin lip injection (Trigeminal inflammatio-n model)	In unmarked cells. Increased evoked excitatory transmission inPBN-CeC synapse.	Right	Miyazawa et al.2018
	Mouse (C57BL/6) *Ex-vivo*	Both	25–68 Days old	CeM	Complete Freund's Adjuvant intoleft hind paw	In unmarked cells. Increased firing in fast spiking phenotype in CeM-PAG projection neurons.	Right	Li and Sheets. 2018
Visceral Pain	Rat (Sprague Dawley) *Ex-vivo*	Male	90–190 g	Not specified	Zymosan-induced colitis	In unmarked cells. Increased firing rateEnhanced PBN-CeA excitatory transmission.	Not specif-ied	Han and Neugebauer, 2004

**Fig. 1:**
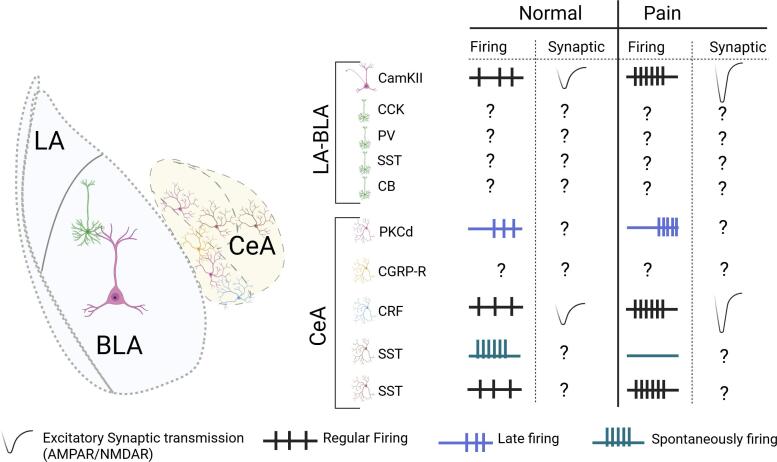
Simplified schematic of cell-types’ firing rate, excitatory synaptic transmission in normal and pain conditions. Abbreviations: CamKII- Calmodulin Kinase II, CCK- Cholecystokinin, PV- Parvalbumin, CB- Calbindin, CR- Calretinin, PKCd-Protein kinase C delta, CGRPR-Calcitonin Gene Related Peptide Receptor, CRF-Corticotropin releasing factor, SST- Somatostatin BLA − Basolateral amygdala, CeA- Central amygdala, CeC- Capsular nuclei of CeA, CeL- Lateral nuclei of CeA, CeM − Medical nuclei of CeA, LA- Lateral amygdala.

Of the widely studied cell types in the amygdala, there remains to be determined, within the context of pain, firing properties and synaptic transmission in CCK, PV, SST and CB neurons in the LA-BLA complex. Within the CeA, synaptic transmission properties of CGRPR, PKCδ and SST neurons and the firing properties of CGRPR neuron are yet to be explored (Fig. 1). Further studies are needed to determine the cell-type specific projection targets, presynaptic inputs for both the major cell-type markers studied widely and emerging newer cell type classifications, thereby, actionable insights can be obtained for identifying therapeutic targets to centrally treat chronic pain mediated affective disorders.

## 5 Physiology of amygdala neurons in the left and Right hemispheres

The role of amygdala, a bilateral brain structure, is often studied under the assumption that the two hemispheres have the same function in pain. Studies over the last two decades have found asymmetrical hemispheric functionality of amygdala in nociception in multiple, but not all, circumstances as reviewed in Allen et al. 2021. A human neuroimaging *meta*-analytical study that investigated amygdala activation in response to various emotional stimuli suggested amygdala functional laterality in terms of temporal dynamics. The right amygdala exhibited a short-term activity while the left showed sustained activity (Sergerie et al. 2008). Rats that were under predatorial stress showed potentiated right and a depressed left amygdala (Adamec et al. 2005). The stress-induced plasticity mechanism is NMDAR-mediated in the right, while non-NMDAR-mediated in the left amygdala. This suggests a lateralized plasticity mechanism in the left and the right amygdala.

Although the relatively unprocessed pathway that carries the nociceptive information through the spino-parabrachial-amygdala pathway equally connects to the left and the right CeA, a unilateral injury causes both right and the left CeA to be activated in adult male rats (Ji et al. 2009). Moreover, in inflammatory pain in male rats, regardless of the side of injury, the right PBN-CeA synaptic transmission is potentiated (Miyazawa et al. 2018) and is dependent on the presence of the neuropeptide CGRP (Shinohara et al. 2017). CGRP-mediated synaptic plasticity in the right CeA showed enhanced NMDA-mediated EPSCs. A similar observation has been made with arthritic pain where pain-induced plasticity in the right CeA is dependent on NMDA and CGRP (Fu et al., 2008, Han et al., 2005). Moreover, in zymosan-induced colitis, a visceral pain model, increased neuronal excitability and PBN-CeA synaptic transmission were observed in the right CeA of male rats (Han et al. 2004). In a peripheral bladder pain stimulation experiment, during an obnoxious urinary bladder distension bout, optogenetic activation of the right CeA increased the pain-related physiological effect in female mice (Sadler et al. 2017). This increase in functional output was replicated after application of PACAP (Pituitary adenylate cyclase-activating polypeptide) *in vivo* on the right side. Activation of the left CeA with optogenetics reduced pain-related physiological effect and reduced mechanical hypersensitivity but PACAP had no effect in the left CeA. Moreover, similar observations were made when PBN^CGRP^ CeA synapses were optogenetically activated in female mice (Allen et al. 2023). This suggests that the left PBN-CeA is involved in an ongoing analgesic activity that is CGRP dependent (Allen et al. 2023). In that study though, CGRP application to naïve mouse slices showed an identical excitatory effect of CGRP on the left and right side of the CeA. Similarly, in a neuropathic pain model, the right CeA activity is observed to be increased by facilitation of PBN-CeA synaptic transmission in male and female rats (Ikeda et al. 2007). Neuropathic pain caused by left side nerve injury increases right CeA neuronal activity at the PBN-CeA synapse in rats (Gonçalves and Dickenson, 2012, Ikeda et al., 2007), however this enhancement is NMDA-receptor independent (Ikeda et al. 2007).

## 6 Conclusions

We are of the opinion that nociception is a critical process in the overall pain experience and that nociception serves a crucial role in protecting us from potential harm. Nociceptive information is transmitted to the CNS for processing and the generation of behavioral responses. The brain integrates diverse sensory signals, translating them into the conscious experience of pain, prompting us to withdraw from harmful stimuli, adjust regular behavior to prevent further injury, and regulate learning to avoid future circumstances that could injure us. Understanding nociception is essential for effective pain management. Further research is necessary to fully elucidate the intricate mechanisms underlying nociception and central sensitization, particularly within a crucial brain structure like the amygdala. This knowledge could lead to the development of more effective treatments for chronic pain.

Repeated exposure to pain inputs can heighten the response to subsequent stimuli and contribute to chronic pain conditions, as well as anxiety and depressive disorders. While this sensitization can be problematic, under normal circumstances, it is reversible, subsiding once the underlying injury heals. In the periphery, electrophysiological studies have provided valuable insights into the essential mechanisms underlying nociception. For example, the functional role of gain-of-function mutations in Nav1.7 and Nav1.8 in generating abnormal nociceptor activity in pain pathologies has been informed by electrophysiological investigations (Tanaka et al., 2017, Faber et al., 2012; Faber et al., 2011). Several potential channel-modulatory pharmacological agents are currently being tested for pain intervention (McDougall and O'Brien, 2024).

The LA-BLA complex primarily consists of glutamatergic excitatory neurons, projecting to the CeA and the intercalated cell mass. In contrast, the CeA and intercalated cell mass are predominantly inhibitory. The LA-BLA complex serves as an input nucleus, while the CeA acts as an output nucleus. The output function of projection neurons originating from the CeA is influenced by several other brain regions. Historically, amygdala studies in pain have often focused on broad populations of neurons using electrophysiological and pharmacological approaches. These approaches can overlook crucial nuances in how different cell types contribute to nociception. Recent RNA-seq studies have revealed a far more diverse cell type composition within the amygdala (O'Leary et al. 2022; Wang et al. 2023). These studies have identified over 20 subtypes, including cells expressing *prkcd*, *sst*, *crf*, *cartpt*, *tacl*, *scn4b*, *vdr,* and *nr2f2*.

The role of each cell type in various pain models requires further investigation. The role of descending pain modulatory pathways in nociception warrants additional exploration. These cell types and pathways can amplify or diminish pain signals depending on various factors. A critical shift towards cell-type-specific studies within these pathways is essential for a deeper understanding of pain mechanisms and the development of more targeted therapies.

Despite the identification of numerous genetically defined neuronal populations within the amygdalar nuclei, relatively few studies have focused on characterizing them in the in terms of pain-mediated neuronal physiological modifications. Some of the cell types that have been studied, so far, in the context of pain, include PKCδ, SST, and corticotropin-releasing hormone/factor (CRF)-expressing neurons (Wilson et al., 2019, Hua et al., 2020, Kiritoshi et al., 2024). Electrophysiological approaches have provided valuable insights into the pain-mediated modifications these cell types undergo. Physiological properties of the LA-BLA interneuron population, including CCK, PV, CB, NPY, CR, VIP, and SST neurons, have yet to be characterized in the context of pain. Consequently, the role of inhibitory control in the LA-BLA complex remains to be elucidated. Within the CeA, the role of the peptide CGRP in excitatory synaptic transmission is largely known in unmarked CeA neurons. Also, the synaptic transmission in SST and PKCδ labeled cells has not been fully explored in the context of pain.

For cell-type-specific investigations, the Cre-lox system has proven highly effective. However, the fidelity of the Cre-lox expression system must be rigorously monitored and documented to avoid non-specific labeling of cells, which can lead to inaccurate interpretation of experimental results. By focusing on cell-type-specific investigations, researchers can gain a more comprehensive understanding of the intricate interplay between nociceptive pathways and pain perception. This approach can shed light on the underlying mechanisms of pain and potentially inform the development of more targeted therapeutic interventions. Finally, the field should prepare itself for exploring computational approaches to integrate data across different laboratories to explore how physiological properties of unique cell types influence each other under acute noxious stimulation conditions and after chronic injury. We have recently begun exploring this idea in the CeA using physiological data from PKCδ and SST neurons to produce two-dimensional (Neilan et al. 2021) and three-dimensional (Neilan et al., 2023, Kraeuter et al., 2024) computational models of the amygdala in the context of acute stimulation and damage accumulation during injury.

## 7 Contributions

BP and BJK developed the concept for the manuscript. BP, MINO, and JRS completed bibliographic research for the review. BP and BJK wrote the manuscript. BP, MINO, JRS, and BJK edited the manuscript.

## 8 Use of Generative AI

During the preparation of this work the author(s) used Copilot – provided by UT Dallas, to correct the citation formats for the references, and for suggestions to improve the language in terms of readability in places where complex processes are explained. After using Copilot, the author(s) reviewed and edited the content as needed and take(s) full responsibility for the content of the publication. All citations have been manually checked.

## CRediT authorship contribution statement

**Blesson K Paul:** Writing – review & editing, Writing – original draft, Visualization, Data curation, Conceptualization. **Maria Isabel Nunez-Ordaz:** Writing – review & editing. **Joseph R. Samuel:** Writing – review & editing. **Benedict J. Kolber:** Writing – review & editing, Writing – original draft, Project administration, Funding acquisition, Conceptualization.

## Declaration of competing interest

The authors declare that they have no known competing financial interests or personal relationships that could have appeared to influence the work reported in this paper.
